# Volatile Cues Influence Host-Choice in Arthropod Pests

**DOI:** 10.3390/ani10111984

**Published:** 2020-10-28

**Authors:** Jacqueline Poldy

**Affiliations:** Commonwealth Scientific and Industrial Research Organisation, Health & Biosecurity, Black Mountain Laboratory, Canberra, ACT 2601, Australia; jacqui.poldy@csiro.au; Tel.: +61-2-6218-3599

**Keywords:** volatilome, host–parasite interactions, vector, VOC, allelochemical, allomone, kairomone, non-host volatile

## Abstract

**Simple Summary:**

Many significant human and animal diseases are spread by blood feeding insects and other arthropod vectors. Arthropod pests and disease vectors rely heavily on chemical cues to identify and locate important resources such as their preferred animal hosts. Although there are abundant studies on the means by which biting insects—especially mosquitoes—are attracted to humans, this focus overlooks the veterinary and medical importance of other host–pest relationships and the chemical signals that underpin them. This review documents the published data on airborne (volatile) chemicals emitted from non-human animals, highlighting the subset of these emissions that play a role in guiding host choice by arthropod pests. The paper exposes some of the complexities associated with existing methods for collecting relevant chemical features from animal subjects, cautions against extrapolating the ecological significance of volatile emissions, and highlights opportunities to explore research gaps. Although the literature is less comprehensive than human studies, understanding the chemical drivers behind host selection creates opportunities to interrupt pest attack and disease transmission, enabling more efficient pest management.

**Abstract:**

Many arthropod pests of humans and other animals select their preferred hosts by recognising volatile odour compounds contained in the hosts’ ‘volatilome’. Although there is prolific literature on chemical emissions from humans, published data on volatiles and vector attraction in other species are more sporadic. Despite several decades since the identification of a small number of critical volatiles underpinning specific host–vector relationships, synthetic chemicals or mixtures still largely fail to reproduce the attractiveness of natural hosts to their disease vectors. This review documents allelochemicals from non-human terrestrial animals and considers where challenges in collection and analysis have left shortfalls in animal volatilome research. A total of 1287 volatile organic compounds were identified from 141 species. Despite comparable diversity of entities in each compound class, no specific chemical is ubiquitous in all species reviewed, and over half are reported as unique to a single species. This review provides a rationale for future enquiries by highlighting research gaps, such as disregard for the contribution of breath volatiles to the whole animal volatilome and evaluating the role of allomones as vector deterrents. New opportunities to improve vector surveillance and disrupt disease transmission may be unveiled by understanding the host-associated stimuli that drive vector-host interactions.

## 1. Introduction

Many agriculturally and medically significant diseases are vectored by arthropod pests, which locate and identify their preferred host species using a variety of host-associated stimuli. Temperature, humidity, and visual signals contribute to host seeking [1], but odour cues contained in the profile of volatile organic compounds (VOCs) emitted by the host—the ‘volatilome’ [2]—are the most important signals. Although a wide variety of VOCs comprise the volatilome, often only a small subset of these are significant for arthropod host selection. Those compounds emitted by one species that modify the physiology or behaviour of another species are known as ‘allelochemicals’ [3]. Allelochemicals are classified further by the ecological benefit or detriment they infer on the recipient: Those that attract pest species are ‘kairomones’; those that repel pest species are ‘allomones’.

There is much to be gained by studying animal volatilomes and the allelochemicals they contain. There is an abundance of knowledge on allelochemicals in the human volatilome, particularly the identity of kairomones and their role in attracting anthropophilic mosquitoes [4,5,6,7,8,9,10,11,12,13,14,15,16,17,18]. By contrast, our understanding of the volatilomes of other vertebrates, and their relevance to arthropod–host interactions, is far less mature. Nevertheless, this field is of increasing importance as the relative plasticity or specificity of the feeding habits of arthropod vectors determines the rate of spread of the disease agents they transmit [19,20]. As three-quarters of emerging infectious diseases are zoonoses and/or are vector-borne [21], increasing our understanding of disease transmission has substantial implications for human health. The veterinary impact of ectoparasitic and predatory arthropods is also a significant burden on livestock industries and companion animal husbandry, driving concerns both to animal welfare and the economic productivity of important agricultural industries. Knowledge of volatile allelochemicals creates opportunities to disrupt pest attack and disease transmission by developing repellents or attractants to enhance vector-trapping for surveillance and monitoring, enabling more efficient pest management.

This paper compiles current knowledge on the chemical ecology of mammalian and avian volatilomes, focusing on allelochemicals of relevance to arthropod pests. A comprehensive table is presented for easy reference to volatile chemicals identified from the species considered. A subselection from this total compendium is also included, indicating those VOCs with allelochemical functionality. This latter table is valuable for comparison of known host–arthropod interactions. Although it is possible that the compounds described in this review include some that are erroneously reported in the primary literature, it is nevertheless a valuable catalogue to compare against the human volatilome. These data represent a starting point to explore chemical means of manipulating host seeking by arthropod pests of veterinary and medical significance. Developing our understanding of the volatile profiles from different species may help to define the drivers for host selection by proposing indicators of non-human hosts and non-hosts.

Inclusion of all research that has contributed to our understanding of ecological interactions is beyond the scope of this review and in this sense, the present work does not intend to be exhaustive. Instead, the aim of this paper is to provide an overview of the evidence that pest species use the volatilomes of animals to discriminate host from non-host species and to document the known specific VOCs responsible for mediating these interactions. By identifying and investigating challenges in collecting non-human animal volatilomes, and in identifying bioactive allelochemical VOCs from these odour profiles, this paper cautions inferences and extrapolations about ecological significance of volatile emissions and highlights opportunities to explore knowledge gaps.

## 2. Challenges in Collecting Volatilomes and Identifying Allelochemicals

As with any field of biochemistry, methodology can strongly influence results. When working with VOCs, techniques for sample collection, VOC discrimination, and elucidation of their biological activity can all significantly influence the findings. In studies of animal volatilomes, many approaches follow procedures developed for investigating the human volatilome. Such investigations are driven by an interest in VOCs for applications such as medical diagnosis and monitoring [22,23,24,25], locating entrapped persons [26,27], or forensic purposes [28], and have documented nearly 2000 compounds from the human volatile odour signature [29]. As these methods have been described and reviewed in other publications [29,30,31] the section below focuses on issues relevant to the collection or testing of non-human animal volatilomes, including identification of VOCs and assessing animal odours to establish allelochemical function.

The transient nature of naturally occurring volatilomes and the chemical properties of VOCs make their accurate capture and identification challenging. VOCs originating from different anatomical or functional compartments have non-uniform representation in each, and the contribution of each compartment to the whole-animal volatile profile varies in time and abundance. Some, such as urinary and faecal emissions, flatulence, and eructation, are transient odour sources. Chemical representations may also be manipulated by microbial community composition and activity, diet, seasonal variation, and environmental conditions. Volatile emissions from animal production, such as broiler facilities [32,33,34], swine fattening operations [35], and cattle feedlots or dairy farms [36,37] can substantially contribute to the environmental odour associated with animals. When investigating arthropod–host interactions, it is important we consider this natural complexity and the methods used to interpret volatilomes.

### 2.1. Methods for Volatilome Collection

No universally accepted methods exist for volatilome collection and many different techniques have been used to sample various anatomical compartments. Collecting animal volatilomes is more challenging compared to human sample collection, in large part because non-human animals cannot be instructed to provide samples on-demand. This is most notable for animal breath collection [38].

For human breath collection—a burgeoning field driven by an interest in non-invasive disease diagnostics—samples are acquired using a variety of methods, although most are aimed at collecting the alveolar, or end-tidal portion of breath [24,31]. To selectively capture alveolar breath, which contains volatile metabolites from the blood stream as well as those arising in lung tissue, air is first expelled from the upper airways, before the latter fraction of breath is exhaled into a collection receptacle for subsequent analysis [31]. Alternatively, methods that monitor exhaled gases in real time can sample the requisite breath directly as is expelled [39,40]. Although some direct-sampling strategies have been adapted from human breath-collection methods [41,42,43,44], the expiratory resistance in these systems and prolonged sampling duration may interfere with normal breathing patterns in non-human animals. The absence of standardised approaches or commercially available collection devices for animals means the experience can be distressing for conscious subjects [38]. Consequently, animal breath samples are usually captured using bag collection techniques [45,46] or from anaesthetised subjects. With only a few studies, typically of small sample size, it is difficult to be confident about the extent of biological and analytical variation or the reproducibility of findings using different methods. Furthermore, for many animals, the influence of contaminants from the nasal passage (for example, for obligate nasal breathers such as horses), or from saliva is unknown. Thus, there are limited data on the contribution from breath to animal volatilomes, and a preference for obtaining samples from more readily accessible compartments such as integument and body odours, which can be acquired without the need for elaborate apparatus or techniques. 

To collect volatile compounds released from integument (skin, hair, feathers, etc.), chemicals may be extracted with a liquid solvent phase or captured from the air when they are naturally emitted. Solvent extraction methods are simpler to perform and do not require specialised equipment, but are unlikely to reflect the natural relative distribution and abundance of compounds in the host volatilome [47]. This is predominantly because different solvents achieve extracts that differ qualitatively and quantitatively in their analyte composition according to their chemical properties [48]. For example, relatively non-polar hexane extracts capture 2-hexanol, 3-hexanol, 2-hexanone, and 3-hexanone from chicken feathers, whereas these analytes are absent in solvent extractions performed with more polar diethyl ether [49]. Extracts produced from these two solvents vary significantly in their appeal to *Culex* mosquitoes [50]. Moreover, in addition to capturing volatile constituents, solvent extraction also captures non-volatile chemicals, such as long-chain alcohol and acid compounds that dominate in avian preen oils. Solvent extraction techniques frequently involve pH manipulation and chemical derivatisation (e.g., transesterification), which may alter the relative abundance of VOCs and the overall volatile profile of compounds present compared to the natural volatile emissions. 

Predictably, chemical sampling methods that directly capture compounds released into the environment immediately surrounding the animal (its ‘headspace’) reveal fewer analytes compared to liquid extraction techniques. By selectively trapping volatile chemicals, systems such as solid phase microextraction (SPME), thermal desorption (TD), or other polymer trapping techniques (e.g., SuperQ or Tenax) deliver a more discerning representation of the natural volatilome profile [51,52,53]. For example, Gabiriot et al. compared traditional solvent extraction with SPME and TD methods to explore the musky odour of the blue petrel (*Halobaena caerulea*) [51]. The volatile chemicals collected with gas-phase sampling techniques were well represented by low molecular weight compounds including sulfides, furans, and imidazole. These smaller VOCs—compounds with much greater vapour pressures—will disperse substantially further than the large waxy materials obtained in abundance from feathers by solvent extraction. Similarly, solvent-free gas-phase sampling techniques were used to capture the short-chain saturated and mono-unsaturated aldehydes that give rise to the distinctive citrus-like aroma of crested auklets (*Aethia cristatella*) and whiskered auklets (*A. pygmaea*) [52,53,54].

Given that methods for collecting volatiles from animal species are less well developed and may be more challenging compared with human research, the existing literature does not yet offer a representative reflection of natural non-human animal volatilomes. In the case of breath volatilomes, even if reproducible methods to collect VOCs from animals are developed, these may not be directly comparable with studies conducted with humans because human data are predominantly obtained from alveolar samples whereas collection from non-human animals principally represents whole-breath samples.

### 2.2. Methods for Volatilome Analysis

In addition to the issues of sample collection outlined above, major challenges in comparing volatilomes from different species arise due to variability between analytical methods, instrumentation, and misapplication of standards between studies. Gas chromatography with mass spectrometric detection (GC-MS) is the principal analytical technique that has been used for studying animal volatilomes, as it is suitable for analysing mixed chemical entities at very low abundance (e.g., pictogram levels). A range of specialised GC-MS based techniques have been developed to increase sensitivity and discrimination of VOCs, and these have been reviewed elsewhere [55,56]. However, analyte quantification obtained on one mass analyser cannot be compared to data acquired on different mass analysers. Instrument stability over time, as well as sample stability, need to be assessed for each instrument [57]. Furthermore, analytes tentatively identified from biological samples through mass spectral library matches (e.g., NIST) should be unequivocally confirmed by applying the same chromatographic methods to authentic standards so that retention times and mass spectral features of putative and reference compounds can be confidently compared. Unfortunately, in several studies, identification of chromatographic peaks has been based solely on mass spectral library matches without validation with synthetic materials [45,48,58,59,60,61,62,63,64,65,66,67,68,69,70,71,72,73]. Such studies cannot identify chromatographic features with confidence.

Misidentification based on library searches can occur for many reasons, including co-elution chromatographic peaks, which may result in a mass spectrum reflecting the summation of the peak composition; the compound being absent from the MS library; and MS similarities, particularly for positional isomers of hydrocarbons. Misidentification is a concern when a compound is considered a distinguishing feature of the volatilome. Furthermore, over-identification is problematic. Mass spectral libraries cannot distinguish between optical isomers; a chiral GC-column would be necessary for chromatographic resolution of these. Nevertheless, many studies have reported a specific isomer despite using an achiral column. It may be that physiological or behavioural activity of an acquired optically active reference material has been demonstrated. Rarely have both isomers been compared or the identity of the natural VOC determined. Reducing the dependence on library matches and ensuring verification with chemical standards will improve the confidence of VOC identification within animal volatilomes.

### 2.3. Methods to Determine Allelochemical Function of Volatiles

Identification of specific putative allelochemicals with ecological relevance is investigated using a suite of field- and/or laboratory-based enquiries. For example, the relative appeal of specific hosts to tsetse flies (*Glossina* sp.) in the field can be measured from the ‘forage ratio’, which assesses the proportion of blood feeding taken from the host species in question, relative to its abundance in the environment [74,75]. Analysis of blood meals imbibed by tsetse flies reveals that a narrow selection of ungulate species, including ox (*Bos* *indicus*) and buffalo (*Syncerus* *caffa*) are most favoured, while others such as waterbuck (*Kobus* *defassa*), hartebeest (*Alcelaphus* *buselaphus*), and impala (*Aepyceros* *melampus*), despite being locally abundant, are avoided [76]. Such field-based findings indicate the feeding preference in natural conditions but offer no information about the chemical drivers behind this choice.

At the other end of the spectrum of methods exploring selective feeding behaviours are electrophysiological studies that measure the ability of individual chemicals or volatile mixtures to stimulate neuronal activity in arthropods (electroantennography, EAG). Frequently, electroantennographic detection is coupled with gas chromatography (GC-EAD) to determine which VOC components in a natural mixture stimulate activity, with mass spectral information typically collected in a separate step by matching retention indices of chromatographic features. A shortfall in these studies is that while a neuronal response signifies the presence of appropriate molecular machinery to recognise and perceive a compound, it gives no information about the behavioural role these VOCs may play. 

A range of behavioural studies are used to elucidate the ecological chemistry at play, to determine if a host-derived chemical acts as an attractant (kairomone) or repellent (allomone) for a given arthropod. These behavioural tests may take the form of laboratory-based choice assays, using apparatus such as Y-tube olfactometers, or field-based lures and traps. Caution must be used in interpreting results for individual compounds assessed in laboratory studies, which might stimulate attraction of the test species compared to a neutral control. Although this provides preliminary evidence for a VOC’s ecological role—assuming it is a natural component of the host’s volatilome—in isolation from a range of other cues, it is difficult to determine if this behaviour replicates the response to the natural host. On the other hand, a lack of response to an isolated chemical is not sufficient to dismiss it as unimportant in host seeking. Currently, neither electrophysiological nor behavioural studies that assess individual chemicals or synthetic mixtures replicate the complexity of a natural host, which includes olfactory interactions and synergistic non-VOC signals such as temperature, humidity, and visual cues.

When neither electrophyisiological nor behavioural response is tested, the identity of allelochemicals within a volatilome sample has sometimes been based solely on conjecture from differing abundance in host vs. non-host species or extrapolated from the behavioural role that specific animal VOCs play in other host–pest relationships. For instance, Douglas et al. suggest that as octanal and hexanal stint bug secretions are potent repellents against invertebrate threats, their presence in crested auklet feathers also serves to fumigate nest sites from ectoparasites [53]. While possible, this assertion lacks evidence. In other research, when behavioural response to synthetic chemicals is documented without regard to the presence or absence of these compounds in the host volatilome [77], the ecological significance of these findings is questionable.

## 3. Known Mammalian and Avian Volatiles and Allelochemicals

There is ample evidence that kairomone and allomone VOCs create an olfactory landscape that vectors navigate in locating a suitable blood source [78,79,80]. Across all the reviewed literature, almost 1300 compounds have been reported as volatiles from non-human mammalian and avian species (Supplementary File 1). No chemical has been reported in all species reviewed. Indeed, half (52%) have only been detected from an individual species, and only a quarter (26%) of the compounds from non-human animal volatilomes have also been recorded from the human volatilome [29]. It is likely that the compounds in Supplementary File 1 will contain inaccuracies arising from the challenges described previously, and it is certainly far from complete.

This list of volatiles in Supplementary File 1, grouped by chemical class (acids, esters, aldehydes, ketones, alcohols, phenols, ethers and furans, hydrocarbons, nitrogen-containing, sulfur-containing, halogenated, and miscellaneous) can be navigated by considering the relevant CAS number for a compound of interest. Although much of the primary literature does not contain CAS numbers, their use should be encouraged to avoid ambiguity arising from non-IUPAC naming conventions, and they have been included where available. Another way to consider these data is to search for an analyte by chemical formula or molecular mass, which may be of value for researches pursuing possible identities of chromatographic features of interest. A subset of the total 1287 VOCs is presented here (Table 1), detailing those volatiles from common domestic species for which evidence of allelochemical potential in selected arthropod pests has been demonstrated.

**Figure 1:**
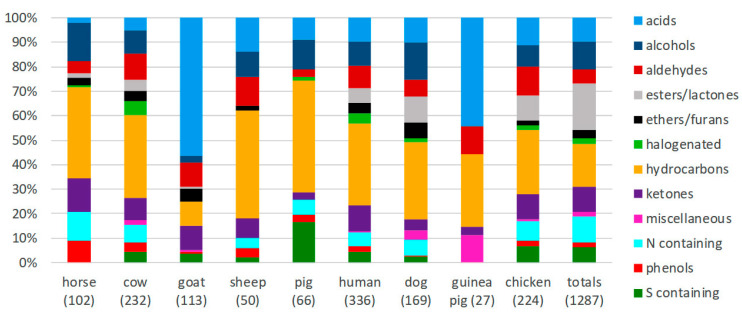
Relative proportion of volatile organic compounds (VOCs) in different chemical classes that have been documented from domestic animal emissions, and across all species reviewed. Numbers in parentheses are the total number of unique compounds reported for the species. Percentages indicate the relative abundance of chemical entities described in each compound class, not their relative concentrations.

While the specific identity of volatile compounds varies, the diversity of VOCs in different chemical classes is similar between species, with hydrocarbons making up a large proportion (Figure 1). The exceptions to this trend are for those species from which data are scarce, where the reported distribution of VOCs may be heavily biased by a small number of studies. For example, records of caprine volatile emissions are swayed significantly by a small number of studies exploring fleece extracts, which are dominated by organic acids, so their emissions may be disproportionately represented by these VOCs [81,82].

Similarly, the data are predisposed to reflect the chemical collection and analysis techniques used. Research with a specific focus or methodology, may over—or under—represent the VOC diversity and chemical class distribution, rather than reflecting the total volatilome composition. For instance, information on avian VOC profiles is based largely on samples collected using solvent extraction of waxy uropygial gland contents and feather materials. The uropygial, or preen gland, is a key source of avian oils, which are applied to feathers to aid water-repellency, thermoregulation, hygiene, and in some instances controlling fungal and bacterial flora [83,84,85,86]. The chemical profiles of these oils are species specific [87] and are predominantly complex mixtures of high-molecular weight esters with characteristic branching patterns and alcohol-acid combinations. Typical esters are composed of C_20_–C_40_ (and up to C_50_) carbon chains [49,50,73,88]. Conversely, almost all odorant chemicals, regardless of their structure or proportions, are compounds with a molecular weight below 300–400Da [89] Although abundant, the physical properties of uropygial and feather waxes means they are unlikely to feature prominently in birds’ volatile odour profiles at ambient temperature. Some authors even suggest that the low volatility of uropygial waxes reduce olfactory conspicuousness to predators [90]. 

Many studies into host chemistry and vector perception have been performed with different agenda, so that either the ecological significance of volatilome components is unknown, or host attraction has not been pursued with rigorous chemical analysis. Research with a specific focus or methodology may misrepresent the compound diversity and chemical class distribution, rather than reflecting the total volatilome composition. The presence of unique compounds in a given volatilome has often been interpreted as implicit evidence for the relevance of these as allelochemicals in vector–host interaction. Without at least electrophysiological data, and ultimately behavioural studies to reinforce these hypotheses, these inferences remain unproven.

Collecting VOCs from animals is significantly more challenging than from human sources. Given this challenge, it is possible that some compounds reported in Supplementary File 1 are not of endogenous origin but arise through sample contamination during collection or storage, through degradation, or changes brought about by measurement/analysis activities, e.g., oxidation or rearrangement with heating. There are significant gaps in our understanding of the origin of many of the VOCs reported, including absences from otherwise continuous homologous series. Are these absences real (and significant), or do they reflect a failure of detection? The occurrence in several studies of compounds known to be plasticisers or material stabilisers needs to be viewed with suspicion as potential contaminants from collection apparatus.

## 4. Animal Volatilomes Influence Host–Pest Interaction

It is well established that odours derived from humans attract insect vectors [91,92]. Similarly, in non-human animals, there is ample evidence for the existence of volatile allelochemical odour clouds that influence interspecies interactions. This section describes examples in which the presence of allelochemicals within host emissions is indicated, but where the identity of the inherent chemicals has not been determined. The following section describes identified allelochemicals within host emissions. 

Despite their limited environmental mobility, several tick species are recognised to exhibit marked host preference. For instance, breeds of domestic cattle that belong to *Bos* *taurus* *taurus* subspecies (e.g., Holstein) suffer significantly greater prevalence and severity of tick-fever compared with *B*. *taurus* *indicus* breeds (e.g., Nelore) and their crosses [93,94]. *B*. *taurus* *indicus* breeds have a high degree of innate resistance to *Babesia* parasites and are marginally more resistant to *Anaplasma* parasites [95,96,97]—both of which can cause tick-fever—transmitted by cattle ticks *Rhipicephalus* (*Boophilus*) *microplus* sensu lato [93,98,99]. While both tick-susceptible and tick-resistant cattle breeds attract tick larvae, significantly more are attracted to olfactory cues in skin rubbings from Holstein calves than those from Nelore calves [100]. Genes that encode enzymes for volatile compound production are expressed at significantly higher levels in susceptible compared with tick-resistant breeds, which may contribute to their increased attractiveness to ticks. In response to biting ticks, an inflammatory response is also exhibited earlier in tick-resistant hosts, correlating with decreased production of tick salivary proteins. Not only do *B*. *taurus* *taurus* cattle develop more debilitating disease when exposed to tick-fever organisms, but they are also at increased risk of exposure as they are more attractive hosts to the tick vectors [100,101,102]. The identity of the attractive or repellent volatiles involved has not been determined. Similarly, brown dog ticks (*Rhipicephalus* *sanguineus*) have markedly different reproductive and development success after feeding on different dog breeds [103,104]. Beagles are unusual in that they are tick-resistant, compared to English cocker spaniels, which are particularly susceptible. Ticks are significantly more attracted to extracts of hair or skin rubbings from tick-susceptible compared to tick-resistant canines [105].

Volatiles cues that provoke differential attraction to specific mosquitoes are well explored, largely in the context of understanding anthropophagic feeding habits amongst a mosaic of alternative host options. Zoophilic and ornithophilic mosquito species also respond selectively to host-derived VOCs. Emissions from avian and bovine blood provoke different attraction and landing responses for female *Aedes* *aegypti* and *Culex* *quinquefasciatus* mosquitoes [4]. These differences mirror the host preferences of zoophilic or ornithophilic species, respectively. Volatile emissions from bacteria originating from human skin attract a significantly higher proportion of *Anopheles* *gambiae* (a highly anthrophophilic species) compared to *An*. *arabiensis* (a generalist species), whereas the inverse is seen for volatiles emitted by chicken skin flora. Equally, while *An*. *gambiae* demonstrates a preference for bacterial species that are strongly associated with humans, *An*. *arabiensis* is uniformly attracted to emissions produced by four diverse bacterial species evaluated, perhaps reflecting the broad range of hosts with their associated microbiota on which it feeds [78]. 

Host-associated microbial volatiles influence other pest arthropods, such as several species of predatory fly. Odours released from mixed-species bacterial communities colonising wounds on warm-blooded animals act as kairomones for screwworm fly species (*Cochliomyia* sp.), which are attracted to and lay their eggs in these lesions [106]. Volatiles released from necrotic tissues are important for advertising oviposition substrates to gravid female screwworm flies [107], which are particularly attracted to tissues already infested with screwworm larvae and wound-associated bacteria [108,109].

As well as odours from live animals and their resident microbial flora, urine and faecal odours and decaying animal remains can also act as arthropod attractants. Tsetse flies are attracted to urine, in particular aged urine, from domestic ox (*Bos* *indicus*), African buffalo (*Syncerus* *caffer*), and bush pig (*Potomochoerus* *porcus*) [110,111]. Faecal waste accumulation increases the attraction of *Aedes* and *Culex* mosquitoes towards live hamsters [112]. Presumably aged waste residues are important to pest species as they indicate reliable host habitat [106]. 

These studies provide abundant evidence that VOCs play as important a role in animal–pest interactions, as they do in human–pest interactions. In these examples, a behavioural response of an arthropod to volatile emissions from a range of animal compartments or animal-associated materials, has been demonstrated. In the following section, evidence that specific chemical analytes act as allelochemicals within these emissions is reviewed.

## 5. Allelochemical VOCs Identified from the Volatilomes of Animals

Carbon dioxide (CO_2_), a ubiquitous respiratory compound, plays important roles in the behaviours of a wide range of arthropods, especially in insect–vertebrate interactions [113]. It is well-established as a kairomone for foraging blood-seeking insects and is particularly effective in luring generalist mosquito species [114,115]. Data from a range of mosquito species and their mammalian hosts indicate that it dictates at least two types of interactions: It *activates* host searching, and, in the presence of other host factors, facilitates *attraction* with orientation and movement towards the source [116]. It also demonstrates a *synergistic* effect when combined with other host odours [117,118].

Octenol (1-octen-3-ol) is another widespread VOC that can be either a kairomone or allomone when released by vertebrates [119]. First identified as an attractant of tsetse flies (*Glossina* sp.)—vectors of African trypanosomiasis—to cattle [120], it is also very effective at attracting other haematophagous insects to hosts that emit it. In combination with CO_2_ and other olfactory cues, it is an important kairomone for zoophilic and anthropophilic mosquitoes such as *Aedes* and *Anopheles* [121,122,123], and female biting midges, *Culicoides* *impunctatus*, and *C*. *nubeculosus* [124], which feed mainly on large mammals such as equids and bovids. Conversely, a lower response or allomonal effect is observed for *Culex* species, which are generally regarded as having ornithophilic feeding preferences [125].

**Figure 2:**
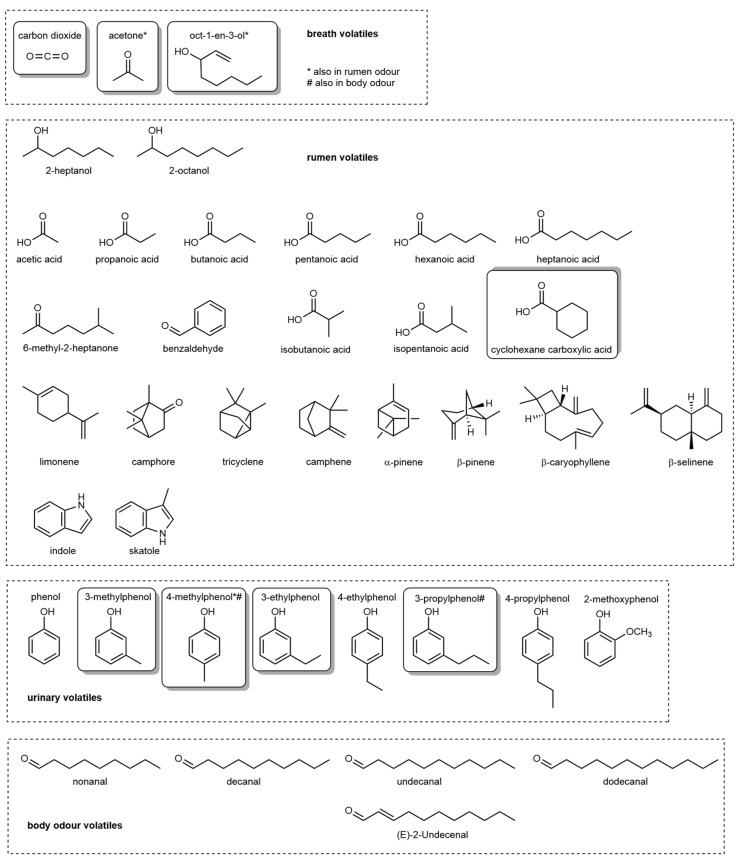
Olfactory attractants of tsetse flies (*Glossina*) from bovine volatile emissions. Broken outlines delineate the different odour compartments from which the analytes are emitted. Solid boxes identify chemical structures that have been shown to demonstrate both electroantennographic and behavioural activity.

In addition to the compounds described above, which are common to a range of host–pest interactions, specific VOCs discussed below, have been shown to be important for certain taxa. Aside from human VOCs, bovine volatiles are probably among the most thoroughly investigated odours in the context of attracting arthropod pests. The area developed from the 1970–1980s’ research on tsetse flies, which formed the groundwork for laboratory and field studies into host finding by chemosensory cues [126]. A 1984 study capturing whole animal emissions from oxen held in tents for up to 80 h collected compounds holistically from skin, breath, rumen eructations, urine, and faeces [120]. Screening total volatile emissions led to the identification of a number of potent tsetse allelochemicals, including CO_2_ [127], acetone [77], and octenol [120,128] in the bovine odour cloud (Figure 2).

Ruminant breath is regularly and frequently contaminated with rumen gases during eructation [129,130]. Metabolic end products that are eructed, such as methane and volatile fatty acids (VFAs), mix with exhaled air when it is expelled via the upper airways. Metabolites that are systemically absorbed from the digestive tract can also be measured in breath as they are eliminated into the airways by blood-gas exchange in the alveoli. Consequently, emanations from breath and rumen gases are often considered together. Despite limited efforts exploring the bovine breath volatilome as an avenue to develop diagnostic tools as indicators of animal health status [45,61,62,63,129,131,132], which follow a parallel path to the flourishing field of human breath analysis [22,23,24,31,133,134,135], few studies have explored animal respiratory emissions as a source of kairomones in arthropod interactions.

**Figure 3:**
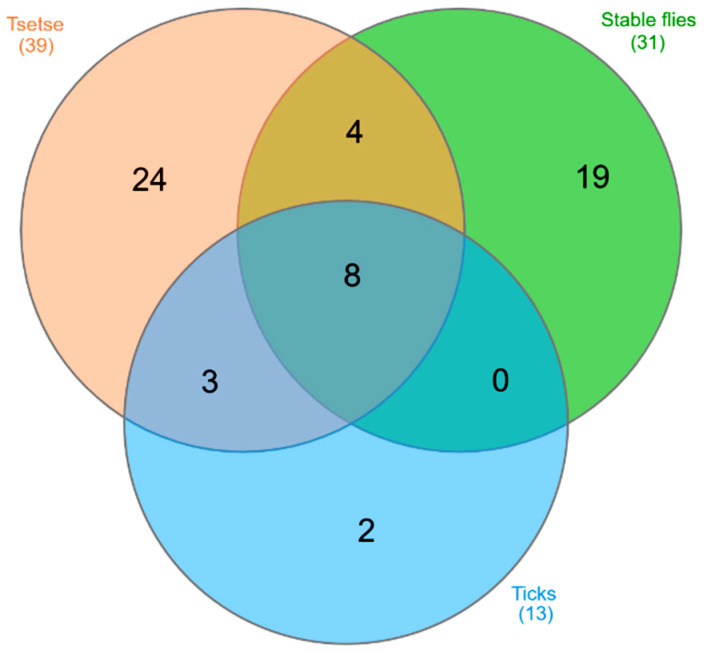
Number of bioactive volatiles from bovine rumen odour that have been shown to stimulate electrophysiological and/or behavioural activity in tsetse fly (39), stable fly (31), and tick (13) species. Numbers in parentheses indicate the total number of allelochemicals in rumen emissions for each pest type; numbers in compartments of the figure show the chemicals with allelochemical function that are shared by different arthropod taxa [138].

Those studies exploring odours associated with rumen fluid show that at least 60 rumen volatiles are attractive to various arthropod pests, including hard tick species [136], tsetse flies [130], and stable flies [137]. Some of these allelochemicals appear to be universally attractive across a range of species, signifying the relevance of rumen eructations to host-seeking arthropods, but others have been noted as attractants in only one or two pest groups (Figure 3). Harraca et al. [130] suggest that the profile of VOCs regularly eructed by ruminants provides chemostimuli that enable tsetse flies to locate their preferred hosts. At least 30 compounds in emissions from bovine rumen contents have been shown to elicit electrophysiological and/or behavioural activity in the stable fly (*Stomoxys* *calcitrans*), a serious pest of large ungulates such as cattle (Table 1). Of these, 4-methylphenol, dimethyl trisulfide, and butanoic acid were noted for their role as kairomones [137].

Unsurprisingly, in their passage through the digestive tract, many of the VOCs that are detectable in rumen fluid also ultimately reach faecal emissions. Odours common to both sources (dimethyl trisulphide, butanoic, acid and *p*-cresol) may act as kairomones to attract tabanids to both oviposition sites (faeces) and nutrition sources (living ruminant host) [137]. Though the two behaviours (feeding and oviposition) rely on attraction to the resource in question (host body and faeces, respectively), it is difficult to establish in laboratory and field studies whether the role of the common chemicals as attractants is relevant to both life history scenarios.

Urine of several ungulate species contains kairomones for many arthropod pests. Tsetse flies are particularly attracted to urinary residues from domestic ox (*B*. *indicus*), African buffalo (*S*. *caffer*), or bush pig (*P*. *porcus*) [110,111], in particular when aged. Unbranched alkyl phenols in these emissions were confirmed through laboratory [139] and field bioassays [139,140] as the attractive components (Figure 2). These kairomones have also been assessed against tabanids flies, including horseflies and deerflies (*Tabanus* *bromis* and *Atylotus* *quadrifarius*), which are large, diurnal, somewhat opportunistic haematophagous insects that are significant livestock pests and vectors of animal pathogens [141,142]. The urinary phenols elicited electroantennographic responses and increased trap catches [141] but in isolation, the synthetic VOCs were less attractive to tabanids than natural sources of ungulate urine [143]. Addition of ammonia synergistically enhanced their attractive properties [142].

Ammonia accumulates in urine over time with aerobic fermentation of urea, accounting for the increased attractiveness of aged urine. Aging of urine also increases the levels of the phenolic attractants themselves [144]. While trace quantities of the free phenols exist in fresh buffalo urine, the majority are present as glucuronate and sulfate conjugates [111]. Specific microorganisms liberate the attractive phenols by hydrolytic activity over time. Sterile urine samples, or those lacking the appropriate microbes, do not yield appreciable quantities of free phenols [144]. This interplay between host kairomones, microbial activity, and insect pests mirrors the changes seen in attractiveness of fresh and aged human sweat samples to *Anopheles* *gambiae* [6]. 

Studies of semiochemical emissions that contribute to territory marking pheromones are well represented from various carnivores [145,146,147,148,149,150,151], but less attention has been given to the existence of allelochemicals in their volatilomes and their role in influencing host location by arthropod pests. Like other mammalian hosts, domestic dogs (*Canis* *lupus* *familiaris*) are susceptible to blood-feeding arthropods and the pathogens they transmit. Extracts of hair and skin rubbings from beagles contained an abundance of analytes that were absent from cocker spaniel extracts, suggesting the differential attraction of brown dog ticks, *R*. *sanguineus*, to different canine races might be due to these differences. Synthetic preparations of the beagle-specific volatiles 2-hexanone and benzaldehyde, and to a lesser extent undecane, acted as non-host allomones to orchestrate avoidance by ticks [152].

Relative to what is known of mammalian volatiles, the chemosensory information contained in the volatilomes of birds has been comparatively underexplored. Campagna and colleagues’ review of avian chemicals highlights a bias for analysis of solvent extractable compounds from the uropygial gland contents and feathers [88], focusing on pheromone-based intraspecific olfactory communication. Fewer studies have investigated allelochemicals driving host–parasite interactions (kairomones or allomones). No doubt practical challenges to collecting volatile odours from avian subjects plays into this, but it is also likely that until relatively recently the significance of avian allelochemicals has been overlooked.

Despite the abundance of low volatility uropygial waxes in oily feather coatings, volatile constituents that can act as long-range allelochemicals are also present. Supporting this are studies demonstrating that chicken feathers or their extracts attract *Ae*. *aegypti*, *Cx*. *quinquefasciatus*, and *Cx*. *nigripalpus* (but not *Cx*. *tarsalis*) in dual-port olfactometer experiments [50]. Although dominated by high molecular weight, non-volatile compounds—the uropygiols [153]—these extracts also contain compounds that are candidates for air-borne mosquito-attractants. These volatile attractants include ketones, alcohols, aldehydes, C_6_–C_9_ carboxylic acids and various saturated and unsaturated hydrocarbons [49,50,73].

On the other hand, the response rate for several mosquito species was substantially greater when presented with a whole live chicken than with isolated feathers, CO_2_, or a combination of feathers and CO_2_ [50]. This suggests a component of avian kairomones must originate from unexplored components of integument and breath, or other compartments. The allelochemical contribution of VOCs from skin of live birds has been largely unexplored. Unlike mammalian hair follicles, feather follicles do not contain glands. Instead, the entire avian skin acts as a sebum-producing holocrine unit, with the potential for odour production and secretion [47].

**Figure 4:**
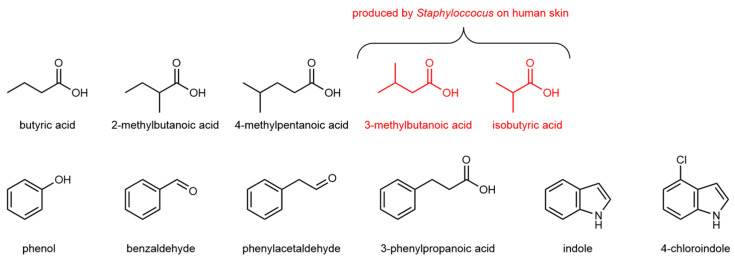
Volatile organic compounds derived from microbial metabolism of hoopoe uropygial secretions [86]. Generation of malodorous fatty acid shows similarities to the compounds produced by cutaneous bacteria on human skin [156].

Odour production in avian species may also involve microbial action on non-volatile integument precursors to produce smaller, more-volatile metabolites [49,88]. The malodorous uropygial secretions of hoopoes (Upupidae) and woodhoopoes (Phoeniculidae) appear to depend on the symbiosis of *Enterococcus* bacteria [154,155]. The volatile emissions include phenolic and indolic compounds, and branched short-chain organic acids similar to those produced by *Staphylococcus* metabolism of human sweat [156] (Figure 4). If administered antibiotics eliminate the birds’ uropygial flora, many of the volatiles contributing to the pungent odours are lost [86]. Although it has not been established whether these modifications influence host-finding behaviour by arthropod pests, the significance of microbial participation in vector attraction has been recognised for the human skin microbiome: The compounds produced by microbial degradation of human sweat elicit physiological and behavioural responses in anthropophilic mosquitoes [6,157,158,159,160].

Crested (*Aethia* *cristatella*) and whiskered auklets (*A*. *pygmaea*) both produce hexanal, hexanoic acid, octanal, and (*Z*)-4-decenal in their volatilomes [54]. Synthetic mixtures of these compounds act as allomones to *Ixodes* *uriae* and *Amblyomma* *americanum* ticks [54], and inhibit landing of the yellow fever mosquito (*Aedes* *aegypti*) [161]. Chewing lice in the genera *Austromenopon* and *Quadraceps* also showed adverse responses when exposed to high concentrations of octanal and (*Z*)-4-decenal. The relevance of these anti-parasitic responses is still uncertain; it has been suggested that the volatile emissions of crested auklets may act as broad-spectrum deterrents against various arthropods [162]. 

Faecal odour is a conspicuous source of volatile compounds from birds [47]. Cooperband et al. (2008) showed in dual-choice experiments that chicken faeces attracted *Culex* *quinquefasciatus* [163]. Mosquito attractant assays and electroantennograms indicated that faecal fractions contained physiologically active compounds including (*E*)-2-decenal, nonanal, undecanal, dodecanal, tetradecanal, pentadecanal, hexadecanal, heptadecanal, and octadecanol. In birds, faecal contamination of nest materials can contribute significantly to the odours emitted from nests, which may be relevant to blood-feeding vectors by way of advertising host habitat [164]. Parent birds frequently perform nest sanitation by removing nestling excrement, which is hypothesised to reduce the intensity of predation, bacterial contamination, arthropod parasitism, and ultimately increase fledgling success [165,166]. Given that avian faeces are recognised as an odour source, it is surprising that few studies have explored the molecular nature of this odour from the perspective of inadvertent signalling to ornithophagic pests.

## 6. How Allelochemicals Influence Host Selection

The above highlights that VOCs in animal volatilomes act as allelochemicals. In this section, the different ways that allelochemicals influence host selection are examined. Allelochemicals can act as kairomones or allomones allowing pest species to fine-tune their feeding choice. The interplay between host-derived chemicals that allows vectors to make specific foraging decisions regarding their preferred host species also permits them to select the individual, and even the body area on which to feed. There is evidence to suggest that some arbovectors are attracted to hosts that are already infected with the agent that they transmit. 

### 6.1. Allelochemicals Used to Distinguish Host and Non-Host Species

Allelochemicals are an important factor in an arthropods’ ability to distinguish and choose a host from a non-host animal. Selection could be driven by the presence of kairomones or the absence of allomones, or the varying levels of both in the volatilome of a potential host. 

A good example is from tsetse flies where electrophysiological studies suggest that the pests may use both attractive aldehydes and a range of deterrents to distinguish between preferred (ox and buffalo) and non-preferred (waterbuck) hosts [76]. Preferred host volatilomes contain medium-chain (C_7_-C_11_) saturated and unsaturated aldehydes, and phenolic components identical to those found in fermented host urine. Although the volatilome of non-preferred waterbuck also contains some of these compounds (4-methylphenol and 3-n-propylphenol), it includes fewer of the tsetse-attractive aldehydes. The non-host volatilome includes compounds that act as allomones: 2-methoxyphenol (guaiacol), 3-isopropyl-6-methylphenol, δ-octalactone, an array of methyl-ketones (C_8_–C_13_), and straight-chain fatty acids (C_5_–C_9_). The difference in body odour between these bovids—creating a kairomonal lure in the case of oxen and buffalos, and an allomonal defense for waterbuck—provides a chemical rationale for host discrimination by tsetse flies.

Certain chemicals can act as either kairomone or allomone for different arthropod species. L-lactic acid is a kairomone for vectors that are anthropophagic, such as the human malaria vector *An*. *gambiae* [10], and *Ae*. *aegypti* [91], but is an allomone for zoophilic and ornithophilic mosquitoes [91] and for tsetse flies, which avoid feeding on humans [167]. The effect of acetone and dimethyl disulfide also depends on the species under investigation: Mosquito response to these human-associated volatiles mirrors their preference for human, other mammalian or avian hosts [91].

### 6.2. Culex Host Switching

Several mosquito species in the *Culex* genus can divert their normally ornithophagic preference to different hosts, based on the availability of the hosts. For instance, when avian hosts migrate away in late summer, *Cx*. *pipiens* turns its feeding bias towards mammals, including humans [168]. Similarly, the feeding preference of *Cx*. *annulirostris* in Australia, which acts as a vector for arboviruses with zoonotic potential (e.g., Ross River, Kunjin and Murray Valley encephalitis viruses), differs depending on regional availability of mammals or birds [73]. It has been suggested that nonanal, a major constituent in the headspace volatiles from human skin, whole chickens, and domestic pigeons (*Columba livia*), and a demonstrated kairomone to *Cx*. *pipiens* *quinquefasciatus*, allows this host-switching behaviour to occur [15].

### 6.3. Allelochemicals That are Used to Isolate Individual Hosts within a Population

The tendency for certain people to be more heavily preyed upon by mosquitos than others is anecdotally well-recognised. Similar intraspecific preferences for individual animals within a population have been identified among other arthropods. Differences in the attractiveness of individual cattle towards haematophagic pests is likely attributable to their VOC phenotype. Holstein-Friesian heifers (*B*. *taurus*) exhibit variable susceptibility towards horn flies (*Haematobia* *irritans*) [169]. Fly-resistant or fly-vulnerable individuals display differences in volatile chemical emissions, with several of these chemicals reported in other studies as allelochemicals governing interactions of haemotophagous pests [170,171].

Some vectors can discriminate the pathogen infection status of individual hosts and apparently choose accordingly. Domestic canaries (*Serinus* *canaria*) that are chronically infected with the avian malaria parasite (*Plasmodium* *relictum*) are more attractive to *Cx*. *pipiens* mosquitoes than uninfected controls. Conversely, birds suffering from acute infection are less attractive [172]. Whether infection-induced changes to the host volatilome profiles have adaptive significance to the malaria parasite in terms of attracting blood-seeking vectors, is uncertain. A previous study of birds exposed to natural malaria infections, in which the duration of the disease for each individual was unknown, reported greater attractiveness of uninfected hosts [173].

Dogs play an important role as reservoirs for *Leishmania* *infantum*, which has significant zoonotic potential, primarily through transmission by sand fly vectors (*Lutzomayia* *longipalpis*). Hair from dogs with leishmaniasis contains a variety of VOCs that are up- or down-regulated compared to uninfected individuals, making them putative biomarkers [174]. Pathogen-induced modification of the host volatilome to facilitate vector transmission has been suggested for golden hamsters (*Mesocricetus* *auratus*), which are more appealing to female sand flies when infected with *L*. *infantum* [175]. Although laboratory assays with synthetic compounds demonstrated activation and attraction of sand flies to the canine VOCs [176], as some of these biomarkers are down-regulated with infection status, the ecological significance of these changes for vector attraction is dubious. Ultimately, evaluating volatilome components out of context from their natural presentation risks misrepresenting their significance as allelochemicals.

Gravid female screwworm flies (*Cochliomyia* sp.) are particularly attracted to tissues that are already infested with screwworm larvae. In this case, it is well established that the change to the volatilome brought about by microbial activity plays an important part in attracting adult females to an appropriate oviposition site [109,177].

### 6.4. Allelochemicals That are Used to Identify Preferred On-Host Feeding-Sites

Some haematophageous pests exhibit on-host feeding-site preferences. The cues that guide the feeding-site orientation are likely to be due to short-range chemicals or changes to the behavioural function of a chemical with local concentration. The highly anthropophilic *An*. *gambiae* selectively bites human feet and ankles, while the opportunistic *An*. *atroparvus* has a propensity to bite around the head and shoulders [9]. Short-chain fatty acids produced by coryneform bacteria found on the skin between human toes acts as a kairomone for *An*. *gambiae* [178] and washing feet with antibacterial soap markedly diverts the biting-site preference of this pest to other body regions. Equally, by excluding exhaled breath, and so removing the main source of CO_2_, the preference of *An*. *atroparvus* for feeding around the head region is significantly diminished [9].

Similarly, tick species with discernible differences in their preferred on-host feeding sites are guided to these regions by attractive odours and repelled from other areas by compounds emanating locally from them. The behaviours of the brown ear tick (*Rhipicephalus* *appendiculatus*) and the red-legged tick (*R*. *evertsi*), which share a geographic range and a common host, are differentiated by their respective feeding sites once on-host. *R*. *appendiculatus* has a predilection for feeding inside the ears of bovine hosts, whereas *R*. *evertsi* is generally located around the anal regions [179]. The ability of adult ticks of these two species to successfully orient and navigate from an alternative place to their usual feeding location on a bovine host, suggests they follow gradients of volatile attractants to the source. Furthermore, when released on-host at the preferred feeding site of the other tick, both species commenced movement to find their favoured destination more rapidly than if released at an otherwise ‘neutral’ location. This indicates that as well as being attracted towards site-specific kairomones, local allomones also act to repel ticks from non-preferred areas. Volatiles collected from the inside of cattle ears and the anal region attracted and repelled *R*. *appendiculatus*, respectively. The opposite effect was observed for *R*. *evertsi*.

There is evidence that VOCs dictate arthropod selection of their host at the level of species, individual, and even feeding site. The interplay between host-derived chemicals that allows vectors to make specific, highly tuned decisions regarding their preferred host species, and also permits them to select the individual and even the body area on which to feed.

### 6.5. Manipulation of Host Parasitism through Application of Synthetic Allelochemicals

Recognising that specific volatile chemical cues drive host selection by arthropod pests suggests opportunities for their artificial application to interfere with host attack. Specifically, the application of allomone compounds identified from non-host odours, can be used to provide an olfactory camouflage to protect otherwise appealing and susceptible hosts. This principal has been most effectively demonstrated in development of a tsetse fly repellent based on VOCs from the non-preferred waterbuck volatilome. A synthetic mixture of geranylacetone, pentanoic acid, guaiacol, and δ-octalactone released from a collar-mounted dispensing system, provided long-term and significant protection to cattle (preferred-hosts) against African trypanosomiasis, vectored by tsetse flies. The large-scale field trial demonstrated significant reduction in disease, and improved bovine health characteristics, which correlated with marked improvement in socioeconomic measures and food security [180]. A similar approach was taken for managing biting black flies (Diptera: Simuliidae) on equine hosts. In this study, a slow-release synthetic preparation of two saturated compounds, C8 and C9 (chemical identities were not provided), mimicking VOC emissions from the European badger, *Meles* *meles*, offered protection to horse ears from black fly attack [181].

## 7. Conclusions

Almost 1300 analytes have been documented in the volatilomes of non-human animals (Supplementary File 1). Studies of volatilome components as they pertain to arthropod–host interactions are limited: Most studies on mammalian chemical profiles have been limited to herbivores and many studies on birds have focused primarily on waxy uropygial secretions, despite evidence that VOCs from other sources may play a greater role as allelochemicals. These gaps in knowledge arise due to challenges in obtaining the samples that contribute to the volatilome, which are exacerbated in non-human subjects. A lack of consistency in volatile-collection methods makes comparisons between studies difficult. In some work, when analysis has relied on chemical libraries without validation using authentic standards, misidentification or overidentification of VOCs is possible, so conclusions need to be interpreted with caution.

It is apparent from the studies reported to date that VOCs originating from different anatomical or functional compartments have non-uniform representation in each. Furthermore, the contribution of each compartment to the whole-animal volatile profile varies. Some, such as urinary and faecal emissions, are transient sources. Chemical representations may also be manipulated by microbial activity, diet, seasonal variation and environmental conditions, and these changes may influence the allelochemical representation and therefore the host appeal. 

To date, most studies of the interaction between arthropod pests and vertebrates focus on the kairomones that actively lure ectoparasites and predatory insects to their preferred hosts, with limited research into allomones. There is a need for greater understanding overall, and especially in the identification of allomones. The possible use of allomones to protect animals and control disease transmission warrants further research in this area.

Several things are clear from the available data. Not all VOCs are physiologically active, and not all physiologically active VOCs are ecologically relevant. By and large we have failed to replicate the attractiveness of natural hosts with any chemical or synthetic mixture of chemicals. The implications of this is that allelochemicals are interaction-specific and complex. These chemicals do not work in isolation in nature and it is most likely that the chemical fingerprint alone, although critical, is not the only stimulus. Other cues such as temperature, humidity, and visual signs contribute to host seeking and selection. It is unlikely that there is a single ‘rule’ governing what constitutes an effective vector-host interaction. We will not identify a single panacea to minimise the impact of arthropod pests on human or animal health and comfort. However, by filling in the gaps for the most significant species—whether from an economic, welfare, or One Health perspective—we can determine where best to focus limited resources.

## Figures and Tables

**Table 1:** A selection of the total (1287) volatile organic compounds (VOCs) collated from the literature from mammalian and avian emissions. The full table (Supplementary File 1) lists and compares the VOCs identified in 141 species across 17 orders. The compounds displayed in this excerpt, from a selection of domestic species, are those with physiological and/or behavioural evidence of allelochemical function in arthropod species of medical or veterinary significance.

Chemical Class	CAS Number	Analyte	Chemical Formula	Molecular Mass	Arthropod Pest(s) for which VOC Displays an Allelochemical Role	Horse (*Equus equus*)	Cattle (*Bos taurus*)	Goat (*Capra aegagrus hircus*)	Sheep (*Ovis aries*)	Pig (*Sus scrofa*)	Human (*Homo sapiens*)	Dog (*Canis familiaris*)	Guinea pig (*Cavia porcellus*)	Rabbit (*Oryctolagus cuniculus*)	Chicken (*Gallus gallus*)
hydrocarbons	1120-21-4	undecane	C_11_H_24_	156.1878	*^1^*	✓	✓			✓	✓	✓	✓		✓
hydrocarbons	629-62-9	pentadecane	C_15_H_32_	212.2504	*^1^*	✓	✓			✓	✓				✓
hydrocarbons	544-76-3	hexadecane	C_16_H_34_	226.2661	*^1^*	✓	✓			✓	✓	✓			✓
hydrocarbons	629-78-7	heptadecane	C_17_H_36_	240.2817	*^1^*						✓				
hydrocarbons	6874-32-4	3,7-dimethyl-(*Z*)-oct-2-ene	C_10_H_20_	140.1565	*^9^*		✓				✓				
hydrocarbons	33501-88-1	2,3,6-trimethylhepta-1,5-diene	C_10_H_18_	138.1409	*^9^*		✓				✓				
hydrocarbons	13828-31-4	1-methyl-3-(1-methylethyl) cyclohexene	C_10_H_18_	138.1409	*^9^*		✓				✓				
hydrocarbons	637-50-3	1-propenylbenzene	C_9_H_10_	118.0783	*^9^*		✓				✓				
hydrocarbons	91-20-3	naphthalene	C_10_H_8_	128.0626	*^1, 9^*		✓				✓	✓			✓
hydrocarbons	138-86-3	limonene	C_10_H_16_	136.1252	*^1, 9^*	✓	✓	✓	✓	✓	✓				✓
hydrocarbons	2436-90-0	citronellene/dihydromyrcene	C_10_H_18_	138.1409	*^9^*	✓	✓		✓		✓				
hydrocarbons	6753-98-6	α-humulene/ α-caryophyllene	C_15_H_24_	204.1878	*^9^*	✓	✓		✓		✓	✓			
hydrocarbons	87-44-5	β-caryophyllene	C_15_H_24_	204.1878	*^9^*	✓	✓		✓	✓	✓				
alcohols	111-70-6	1-heptanol	C_7_H_16_O	116.1201	*^9^*		✓				✓				
alcohols	543-49-7	2-heptanol	C_7_H_16_O	116.1201	*^5^*	✓	✓								
alcohols	111-87-5	1-octanol	C_8_H_18_O	130.1358	*^9^*		✓		✓		✓	✓			
alcohols	123-96-6	2-octanol	C_8_H_18_O	130.1358	*^5^*	✓	✓								
alcohols	589-98-0	3-octanol	C_8_H_18_O	130.1358	*^9^*	✓	✓				✓				✓
alcohols	143-08-8	1-nonanol	C_9_H_20_O	144.1514	*^9^*	✓	✓				✓				
alcohols	112-30-1	1-decanol	C_10_H_22_O	158.1671	*^3^*						✓				
alcohols	1120-06-5	2-decanol	C_10_H_22_O	158.1671	*^9^*		✓				✓				
alcohols	928-96-1	(*Z*)-3-hexen-1-ol	C_6_H_12_O	100.0888	*^9^*		✓								✓
alcohols	928-92-7	4-hexen-1-ol	C_6_H_12_O	100.0888	*^1^*						✓				
alcohols	4938-52-7	1-hepten-3-ol	C_7_H_14_O	114.1045	*^1^*						✓				
alcohols	3391-86-4	1-octen-3-ol	C_8_H_16_O	128.1201	*^2, 4, 5, 7, 8, 9^*	✓	✓				✓				✓
alcohols	123-51-3	3-methyl-1-butanol/isopentanol	C_5_H_12_O	88.0888	*^2^*						✓				✓
alcohols	104-76-7	2-ethylhexanol	C_8_H_18_O	130.1358	*^1, 7, 9^*		✓	✓			✓	✓		✓	✓
alcohols	107-21-1	ethan-1,2-diol/ethylene glycol	C_2_H_6_O_2_	62.0368	*^3^*						✓				
alcohols	56-81-5	1,2,3-propanetriol/glycerol	C_3_H_8_O_3_	92.0473	*^1, 3^*						✓				
alcohols	18479-58-8	dihydromyrcenol	C_10_H_20_O	156.1514	*^1^*						✓	✓			
alcohols	78-70-6	linalool	C_10_H_18_O	154.2530	*^1, 9^*	✓	✓		✓		✓				
alcohols	89-78-1	menthol	C_10_H_20_O	156.1514	*^1^*						✓	✓			
alcohols	106-22-9	dihydrogeraniol/citronellol	C_10_H_20_O	156.1514	*^9^*						✓				
alcohols	57-88-5	cholesterol	C_27_H_46_O	386.3549	*^3^*						✓				
alcohols	100-51-6	benzyl alcohol	C_7_H_8_O	108.0575	*^3^*		✓				✓	✓			✓
phenols	108-95-2	phenol	C_6_H_6_O	94.0419	*^6, 7^*	✓	✓			✓	✓	✓		✓	✓
phenols	108-39-4	3-methylphenol/ *m*-cresol	C_7_H_8_O	108.0575	*^4, 6, 7, 9^*	✓	✓	✓			✓				
phenols	106-44-5	4-methylphenol/ *p*-cresol	C_7_H_8_O	108.0575	*^2, 4, 5, 6, 7, 9^*	✓	✓		✓	✓	✓			✓	✓
phenols	620-17-7	3-ethylphenol	C_8_H_10_O	122.0732	*^4, 5, 7^*		✓								
phenols	123-07-9	4-ethylphenol	C_8_H_10_O	122.0732	*^2, 6, 7^*		✓								✓
phenols	621-27-2	3-propylphenol	C_9_H_12_O	136.0888	*^4, 5, 6, 7^*	✓	✓		✓						
phenols	645-56-7	4-propylphenol	C_9_H_12_O	136.0888	*^7^*		✓								✓
phenols	499-75-2	3-isopropyl-6-methylphenol	C_10_H_14_O	150.1045	*^4^*						✓			✓	
phenols	90-05-1	2-methoxyphenol	C_7_H_8_O_2_	124.0524	*^4, 5, 7, 9^*	✓	✓				✓				✓
phenols	119-33-5	4-methyl-2-nitrophenol	C_7_H_7_NO_3_	124.0524	*^9^*		✓								
acids	64-18-6	methanoic acid/formic acid	CH_2_O_2_	46.0055	*^1^*						✓				
acids	64-19-7	ethanoic acid/ acetic acid	C_2_H_4_O_2_	60.0211	*^1, 3, 5, 9^*		✓	✓	✓		✓	✓			✓
acids	79-09-4	propanoic acid	C_3_H_6_O_2_	74.0368	*^1, 2, 5, 9^*		✓	✓	✓		✓	✓			✓
acids	107-92-6	butanoic acid	C_4_H_8_O_2_	88.0524	*^1, 2, 5, 8, 9^*	✓	✓	✓	✓		✓	✓			✓
acids	109-52-4	pentanoic acid/valeric acid	C_5_H_10_O_2_	102.0681	*^1, 2, 8^*		✓	✓	✓		✓	✓			✓
acids	142-62-1	hexanoic acid	C_6_H_12_O_2_	116.0837	*^1, 2, 3, 8, 9^*		✓	✓			✓	✓			✓
acids	111-14-8	heptanoic acid	C_7_H_14_O_2_	130.0994	*^1, 2, 3, 8^*		✓	✓			✓	✓			✓
acids	124-07-2	octanoic acid	C_8_H_16_O_2_	144.115	*^1, 2, 3^*			✓			✓	✓			✓
acids	112-05-0	nonanoic acid	C_9_H_18_O_2_	158.1307	*^1, 2, 3^*			✓			✓				
acids	334-48-5	decanoic acid	C_10_H_20_O_2_	172.1463	*^1, 2, 3^*			✓		✓	✓				
acids	112-37-8	undecanoic acid	C_11_H_22_O_2_	186.162	*^1, 2, 3^*						✓				
acids	143-07-7	dodecanoic acid	C_12_H_24_O_2_	200.1776	*^1, 2, 3^*			✓		✓	✓				✓
acids	638-53-9	tridecanoic acid	C_13_H_26_O_2_	214.1933	*^1, 2, 3^*			✓			✓				
acids	544-63-8	tetradecanoic acid/myristic acid	C_14_H_28_O_2_	228.2089	*^1^*	✓		✓		✓	✓	✓	✓		✓
acids	1002-84-2	pentadecanoic acid	C_15_H_30_O_2_	242.2246	*^1, 3^*	✓		✓			✓		✓		✓
acids	57-10-3	hexadecanoic acid/palmitic acid	C_16_H_32_O_2_	256.2402	*^1, 2, 3^*	✓	✓	✓		✓	✓	✓	✓	✓	✓
acids	506-12-7	heptadecanoic acid	C_17_H_34_O_2_	270.2559	*^1, 3^*			✓			✓		✓		✓
acids	57-11-4	octadecanoic acid/stearic acid	C_18_H_36_O_2_	284.2715	*^1, 2, 3^*		✓	✓			✓	✓	✓	✓	✓
acids	79-31-2	2-methylpropanoic acid/isobutyric acid	C_4_H_8_O_2_	88.0524	*^5, 8, 9^*		✓	✓	✓		✓	✓			✓
acids	503-74-2	3-methylbutanoic acid/isovaleric acid	C_5_H_10_O_2_	102.0681	*^1, 2, 5, 9^*		✓		✓		✓	✓			✓
acids	27960-21-0	(*E*)-3-methyl-2-hexenoic acid	C_7_H_12_O_2_	128.0837	*^2^*						✓				
acids	18719-24-9	7-octenoic acid (+CO_2_)	C_8_H_14_O_2_	142.0994	*^2^*						✓				
acids	50-21-5	2-hydroxypropanoic acid/lactic acid	C_3_H_6_O_3_	90.0317	*^1, 2, 3, 6, 7^*						✓				
acids	98-89-5	cyclohexanecarboxylic acid	C_7_H_12_O_2_	128.0837	*^5^*		✓				✓				
esters	698-76-0	*δ*-octalactone	C_8_H_14_O_2_	142.0994	*^4, 5^*										
esters	105-66-8	propyl butanoate	C_7_H_14_O_2_	130.0994	*^9^*		✓				✓				✓
acids	65-85-0	benzoic acid	C_7_H_6_O_2_	122.0368	*^1, 3, 8^*			✓			✓	✓			✓
aldehydes	123-38-6	propanal	C_3_H_6_O	58.0419	*^3^*						✓				✓
aldehydes	66-25-1	hexanal	C_6_H_12_O	100.0888	*^1, 8^*		✓	✓		✓	✓	✓	✓		✓
aldehydes	111-71-7	heptanal	C_7_H_14_O	114.1045	*^1, 3, 7^*		✓	✓	✓		✓	✓			✓
aldehydes	124-13-0	octanal	C_8_H_16_O	128.1201	*^1, 4, 7^*		✓	✓	✓		✓	✓			✓
aldehydes	124-19-6	nonanal	C_9_H_18_O	142.1358	*^1, 3, 4, 7^*	✓	✓	✓	✓		✓	✓	✓	✓	✓
aldehydes	112-31-2	decanal	C_10_H_20_O	156.1514	*^1, 4, 7, 9^*	✓	✓	✓	✓		✓	✓		✓	✓
aldehydes	112-44-7	undecanal	C_11_H_22_O	170.1671	*^4^*	✓	✓				✓			✓	✓
aldehydes	112-54-9	dodecanal	C_12_H_24_O	184.1827	*^1, 4^*	✓	✓				✓			✓	✓
aldehydes	18829-55-5	(*E*)-2-heptenal	C_7_H_12_O	112.0888	*^5, 4^*		✓				✓				✓
aldehydes	18829-56-6	(*E*)-2-nonenal	C_9_H_16_O	140.1201	*^1, 5, 7^*	✓	✓	✓			✓				
aldehydes	53448-07-0	(*E*)-2-undecenal	C_11_H_20_O	168.1514	*^4^*		✓				✓				
aldehydes	432-25-7	β-cyclocitral	C_10_H_16_O	152.1201	*^9^*		✓								
aldehydes	100-52-7	benzaldehyde	C_7_H_6_O	106.0419	*^1, 5^*		✓	✓	✓		✓	✓	✓	✓	✓
aldehydes	122-78-1	phenylacetaldehyde	C_8_H_8_O	120.0575	*^3, 7^*		✓	✓	✓		✓				
ketones	67-64-1	acetone	C_3_H_6_O	58.0419	*^1, 2, 6, 7^*		✓	✓	✓		✓	✓			✓
ketones	78-93-3	butanone	C_4_H_8_O	72.0575	*^1, 6, 7^*		✓	✓			✓				✓
ketones	107-87-9	2-pentanone	C_5_H_10_O	86.0732	*^1^*		✓	✓			✓				✓
ketones	96-22-0	3-pentanone	C_5_H_10_O	86.0732	*^1^*						✓				✓
ketones	110-43-0	2-heptanone	C_7_H_14_O	114.1045	*^9^*	✓	✓	✓			✓	✓			✓
ketones	111-13-7	2-octanone	C_8_H_16_O	128.1201	*^4, 5^*	✓					✓				
ketones	106-68-3	3-octanone	C_8_H_16_O	128.1201	*^9^*	✓	✓	✓			✓				
ketones	821-55-6	2-nonanone	C_9_H_18_O	142.1358	*^5, 4^*	✓					✓				
ketones	693-54-9	2-decanone	C_10_H_20_O	156.1514	*^1, 4^*						✓				
ketones	112-12-9	2-undecanone	C_11_H_22_O	170.1671	*^9, 4^*		✓				✓				
ketones	6175-49-1	2-dodecanone	C_12_H_24_O	184.1827	*^4^*		✓				✓				
ketones	110-93-0	6-methyl-5-hepten-2-one	C_8_H_12_O	124.0888	*^1, 2, 7, 9^*	✓	✓	✓	✓		✓	✓			✓
ketones	3796-70-1	(*E*)-geranylacetone	C_13_H_22_O	194.1671	*^1, 2, 4, 5, 9^*		✓				✓	✓			
ketones	7764-50-3	dihydrocarvone	C_10_H_16_O	152.1201	*^9^*		✓								
ketone	76-22-2	camphor	C_10_H_16_O	152.1201	*^5^*		✓				✓	✓			
ketones	98-86-2	acetophenone	C_8_H_8_O	120.0575	*^9^*	✓	✓				✓	✓		✓	✓
amines	7664-41-7	ammonia	NH_3_	17.0265	*^1, 2^*		✓				✓				✓
amines	120-72-9	indole	C_8_H_7_N	117.0578	*^1, 2, 5^*	✓	✓		✓	✓	✓	✓			✓
amines	83-34-1	3-methylindole/skatole	C_9_H_9_N	131.0735	*^5, 9^*	✓	✓		✓	✓	✓				✓
halogenated	75-09-2	dichloromethane	CH_2_Cl_2_	83.9534	*^1^*						✓				✓
halogenated	106-46-7	1,4-dichlorobenzene	C_6_H_4_Cl_2_	145.969	*^1^*						✓				✓
sulfides	75-18-3	dimethyl sulfide	C_2_H_6_S	62.019	*^1^*		✓	✓			✓				✓
sulfides	624-92-0	dimethyl disulfide	C_2_H_6_S_2_	93.9911	*^1^*		✓	✓			✓	✓			✓
sulfides	110-81-6	ethyl disulfide	C_4_H_10_S_2_	122.0224	*^1^*										✓
sulfides	2179-60-4	methyl propyl disulfide	C_4_H_10_S_2_	122.0224	*^1, 3^*						✓				
sulfides	3658-80-8	dimethyl trisulfide	C_2_H_6_S_3_	125.9632	*^1, 3, 5, 9^*		✓	✓	✓	✓	✓				✓
sulfides	75-15-0	carbon disulfide	CS_2_	75.9441	*^1^*		✓			✓	✓				✓
thiazole	95-16-9	benzothiazole	C_7_H_5_NS	135.0143	*^2^*		✓				✓	✓			

^1^*Aedesaegypti*. ^2^*Anophelesgambiae*. ^3^*Culexquinquefasciatus*. ^4^*Glossinamorsitans*. ^5^*Glossinapallipides*. ^6^*Culicoidesimpunctatus*. ^7^*Culicoidesnubeculosus*. ^8^*Rhipicephalusmicroplus*. ^9^*Stomoxyscalcitrans*.

## Supplementary Materials

The following is available online at https://www.mdpi.com/2076-2615/10/11/1984/s1, Supplementary File 1: All 1287 volatile organic compounds reported in the literature from 141 non-human animal species. References [182,183,184,185,186,187,188,189,190,191,192,193,194,195,196,197,198,199,200,201,202,203,204,205,206,207,208,209,210,211,212,213,214,215,216,217,218,219,220,221,222,223,224,225,226,227,228,229,230,231,232,233,234,235,236,237,238,239,240,241,242,243,244,245,246,247,248,249,250,251,252,253,254,255,256,257,258,259,260,261,262,263,264,265,266,267,268,269,270,271,272,273,274,275,276,277,278,279,280,281,282,283,284,285,286,287,288,289,290,291,292,293,294,295,296,297,298,299,300,301,302,303,304,305,306,307,308,309,310,311,312,313,314,315,316,317,318,319,320,321,322,323,324,325,326,327,328,329,330,331,332] are cited in the supplementary materials.
